# Intestinal parasitic infections in children from marginalised Roma communities: prevalence and risk factors

**DOI:** 10.1186/s12879-024-09500-z

**Published:** 2024-06-18

**Authors:** Gabriela Štrkolcová, Daniela Fiľakovská Bobáková, Michaela Kaduková, Andrea Schreiberová, Daniel Klein, Miloš Halán, Ingrid Urbančíková

**Affiliations:** 1 Department of Epizootiology and Parasitology and Protection of One Health, University of Veterinary Medicine and Pharmacy in Košice, Košice, Slovakia; 2 Department of Health Psychology and Research Methodology, Faculty of Medicine, PJ Safarik University, Kosice, Slovakia; 3 Olomouc University Social Health Institute, Palacky University in Olomouc, Olomouc, Czechia Czechia; 4 Institute of Mathematics, Faculty of Natural Sciences, PJ Safarik University, Kosice, Slovakia; 5 Department of Epidemiology, Faculty of Medicine, PJ Safarik University, Kosice, Slovakia

**Keywords:** Parasitic infections, Risk factors, Hygienic standards, Un-dewormed animals, Health complaints, Marginalised Roma communities, Early childhood

## Abstract

**Background:**

Intestinal parasitic infections remain a significant global health issue, particularly affecting poor and marginalised populations. These infections significantly contribute to children’s diseases, malnutrition, poor school performance, cognitive disorders, and future economic losses. This study aimed to explore and compare the occurrence of intestinal parasites in early childhood among the group of infants from the Slovak majority population and from marginalised Roma communities (MRCs). Furthermore, it aimed to explore the health complaints of children with and without intestinal parasitic infection in the past month and assess the effect of various risk factors on the occurrence of intestinal parasitic infection in infants from MRCs.

**Methods:**

We obtained cross-sectional data from mothers and stool samples of their children aged 13–21 months using the first wave of the longitudinal RomaREACH study. A total of 181 stools from infants were analysed: 105 infants from the Slovak majority population and 76 from MRCs.

**Results:**

Infants from MRCs are significantly more often infected by *Ascaris lumbricoides*, *Trichuris trichiura* and *Giardia duodenalis* than their better-off peers from the majority population. Infection rates are 30% in infants from MRCs vs. 0% in the majority population (*p* < 0.001). Single and mixed infections were observed in children from MRCs. Infants with intestinal parasitic infections suffer significantly more often from various health complaints, particularly cough, stomach ache, irritability, and diarrhoea. Within MRCs, the risk of parasitic infections in infants is significantly increased by risk factors such as the absence of flushing toilets in households (OR = 4.17, *p* < 0.05) and contact with un-dewormed animals (OR = 3.61, *p* < 0.05). Together with the absence of running water in the household, these three factors combined increase the risk more than ten times (*p* < 0.01).

**Conclusion:**

Maintaining hygienic standards in conditions of socioeconomic deprivation in MRCs without running water and sewage in the presence of un-dewormed animals is problematic. These living conditions contribute to the higher prevalence of parasitic infections in children from MRCs, causing various health complaints and thus threatening their health and healthy development.

**Supplementary Information:**

The online version contains supplementary material available at 10.1186/s12879-024-09500-z.

## Introduction

Intestinal parasitic infections caused by enteric protozoa and helminths still represent a significant global health problem despite the efforts that have been increasingly exerted over the last decades to promote good hygiene and sanitation in the exposed regions. The afflicted communities include the individuals who are most vulnerable, especially poor and marginalised populations in areas with low hygiene and economic standards. Such infections negatively affect health and life, particularly for children from the poorest parts of the world, and have extensive financial and social consequences [1, 2]. Marginalised Roma communities (MRCs) are among the most disadvantaged and underserved communities in the EU. In Slovakia but also elsewhere, people living in MRCs face discrimination and socioeconomic deprivation, have limited access to health care [3] and have low health literacy [4]. Roma households are often overcrowded and lack indoor tap water or toilets, which makes following hygiene and prevention recommendations difficult for household members as well as whole communities [3].

Globally, intestinal parasitic infections affect an estimated 3.5 billion individuals, with 450 million experiencing symptomatic illness and an annual mortality rate exceeding 200,000 deaths [5, 6]. Most intestinal infections are caused by protozoan parasites *Giardia duodenalis* and helminths, such as *Ascaris lumbricoides, Trichuris trichiura* and *Hymenolepis* spp. According to the World Health Organisation (WHO) statistics, more than a quarter of the global population in developing countries suffer from those parasitoses [7].

*Giardia duodenalis* is a cyst-forming intestinal parasite that settles in the mucosa of the small intestine in humans and animals. Two genetically different groups (assemblages), A and B, are of public health importance and are often associated with outbreaks of epidemics originating in water and foods. *G. duodenalis* is one of the most frequently occurring protozoan parasites that cause diarrhoea in humans all over the world, with approximately 280 million symptomatic individuals and 500 thousand new cases reported every year [8, 9]. In many countries, giardiasis is a reportable disease. *Giardia* is one of the four most frequent enteropathogens that afflict children at an early age, i.e. less than two years, and cause weight loss, malabsorption, stunting, and disruption to cognitive development [10–12]. *Giardia* cysts are resistant in the outdoor environment and can survive for several weeks in soil and surface water [13].

The species from the *Cryptosporidium* genus are classified as intracellular coccidia with a cosmopolitan distribution. Cryptosporidiosis is a common gastrointestinal disease in children aged 1 to 5 years in developing as well as developed countries [14, 15]. The protozoan *Cryptosporidium* is a leading cause of diarrhoea morbidity and mortality in children younger than five years. Khalil et al. 2018 and Pumipuntu et al. 2018 [16, 17] confirmed that in 2016, cryptosporidiosis was the fifth most frequent cause of diarrhoea syndrome in children younger than five years and has caused more than 48,000 deaths all over the world. Giardiasis and cryptosporidiosis afflict approximately 2.4 billion people, while the most vulnerable group of children includes those aged 2 to 5 years [7].

Soil-transmitted helminths (STHs), also referred to as geohelminths, primarily afflict children who live in extreme poverty and predominantly also those who live in rural areas or communities with a lack of water, poor hygiene and low education [1, 23, 18]. Although patients with low-intensity infections caused by STHs may be asymptomatic, moderate- to high-intensity infections are associated with gastrointestinal symptoms, anaemia, as well as deterioration of the physical development of children [19–21]. According to the WHO, STHs infect approximately 1.5 billion people globally. Almost two billion people, i.e. nearly a fourth of the total global population, become infected by one or more species of STHs, most frequently all by roundworm *Ascaris lumbricoides*, whipworm *Trichuris trichiura* and tapeworms, of which *Hymenolepis nana* is globally the most frequent tapeworm infecting the human population [2, 22].

*Ascaris lumbricoides* is the most frequent nematode infecting the small intestine of humans with a cosmopolitan distribution. Ascariasis is the most common helminthiasis in the world and occurs primarily in sub-Saharan Africa, Latin America, China and the Indian subcontinent [2, 18, 23]. Ascariasis negatively affects the nutrition and cognitive development of preschool and school children [2, 24, 25]; the most vulnerable group comprises preschool-aged children [26, 27]. Females have an excellent reproduction ability – they lay 200,000 eggs per day, hence intensively contaminating the environment [28, 29]. When an infectious egg is ingested, it releases a larva that migrates into the host’s body. During pulmonary migration, it causes pneumonia. Occasionally, larvae enter the pancreatic and bile ducts, where they cause blockage, and hence reduce the lactose metabolism and cause vitamin A deficiency, which may lead to retardation of growth, malnutrition and cognitive disorders in children [23, 30, 31]. Prevalence of ascariasis in tropical and subtropical regions ranges from 70 to 90%, with an average incidence of 446 million cases per year, whereas in Europe, it amounts to 0.5–24.7%. Despite such a high prevalence, ascariasis ranks among neglected tropical diseases negatively impacting public health [32–35].

*Trichuris trichiura* is the whipworm that infects 795 million people [22]. According to WHO, around 800 million people worldwide are affected by trichuriasis, including 114 million preschool-aged children and 350 million school-aged children [2, 36–38]. The life cycle is direct; after ingesting infectious larvated eggs from contaminated soil, water or food, the eggs hatch in the large intestine moult four times to adults. The female can excrete 3000–5000 typical lemon-shaped eggs into the external environment. Adult worms can survive in the intestine for an extended period, approximately ten years or longer [22, 39]. Depending on the number of worms, infections can be without symptoms. Higher-intensity infection can present with abdominal pain, nausea, vomiting, signs resembling appendicitis, fever, headache, weight loss, and anaemia. Severe infections can lead to bloody diarrhoea, weight loss, dehydration, hypochromic anaemia, and potentially death [14, 22, 40, 41]. Faeces contain mucus and sometimes also blood. Massive trichuriasis in children, referred to as Trichuris Dysenteric Syndrome - TDS, is characterised by abdominal pain, tenesmus, and anal rectal prolapse [22, 42, 43].

*Hymenolepis nana* is the most common tapeworm, commonly known as the dwarf tapeworm, and it infects people and various rodents. *H. nana* occurs cosmopolitanly and is considered the most common cause of cestodiasis in humans, especially children and individuals living in poor hygiene conditions [44]. Definitive hosts become infected through the oral ingestion of eggs in contaminated water, food and soil. From an epidemiological perspective, it is important to note that various species of rodents also serve as hosts, acting as reservoirs for the disease and contributing to its spread within the human population. The risk arises from both the high population density of rodents near human dwellings and the presence of rodents in areas where food is stored or processed, leading to the potential contamination of food with eggs from the faeces of infected animals [45, 46].

Over the last decade, several studies have been conducted to investigate the impact of STHs on children’s health and cognitive functions [18, 20, 23, 24, 47, 48]. Even though infections caused by intestinal parasites are not among the leading causes of death of children at the global level, they are important causes of children’s diseases, malnutrition, poor school performance, cognitive disorders, and consequently also, future economic losses [20, 49, 50].

The aim of this study was to explore and compare the occurrence of intestinal parasites in early childhood in the group of infants in marginalised Roma communities (MRCs) with the occurrence in infants in majority communities and to assess the contribution of risk factors (no running water in the household, no flushing toilet and contact with undewormed animals) to higher occurrence of parasites in such MRCs.

## Materials and methods

### Study area

We used data from the first wave of the longitudinal RomaREACH study. The data were collected in 2021–2022 in the Prešov and the Košice regions of Slovakia. For the present study, respondents for whom a stool sample of the child was available were included in the analyses. Our sample consisted of 181 children aged 13–21 months (median [IQR]: 16 [15–17]). We included two populations in the sample: 76 children from marginalised Roma communities and 105 children from the Slovak majority population. Participants were recruited in three ways: (1) via paediatricians during regular preventive check-ups (mother-child dyads from both populations), (2) via Roma health mediators and social workers directly in the communities (mother-child dyads from MRCs), (3) via parental groups on social media (mother-child dyads from the majority).

Data used for the analyses were obtained by self-report questionnaires and stool samples collected from the children during mandatory preventive paediatric visits, at the community centres and in the respondents’ households. Mothers were provided with plastic containers and asked to collect the stool samples no more than 24 h before the pick-up. They were instructed to store it in the refrigerator until the visit to the primary paediatricians for preventive check-ups or arranged pick-ups of the samples by research team members. Containers were labelled with a unique code corresponding to the database code, including children’s data and the collection date. Samples were transported in a polystyrene thermal box for transporting biological material to the Department of Epizootiology and Parasitology and Protection of One Health, University of Veterinary Medicine and Pharmacy in Košice for further processing. Signed informed consent was obtained from the mothers of all participating children. Participation in the study was fully voluntary and confidential. The Roma REACH study was approved by the Ethical Committee at the Faculty of Medicine, University of Pavol Jozef Šafárik in Košice (No. 16 N/2021) and by the Ethical Committees of Košice and Prešov Self-governing Region (under “RomaREACH” and No. 03682/2022/OZ-20, respectively).

### Measures

We assessed a series of sociodemographic variables and risk factors for the occurrence of parasitic diseases. Items from the questionnaire can be found in Appendix 1.

Residence in a marginalised Roma community as a determinant variable was assessed based on the Atlas of Roma Communities - the sociographic mapping conducted by the Office of the Government Plenipotentiary for the Roma Communities in cooperation with the Institute for Work and Family Research undertaken in 2019 [51] as well as by the verification the question “Are your closest neighbours mostly Roma?”

To assess the level of maternal education [52], we used the question: “What education have you completed? (Elementary school, Apprentice school, Secondary school, University).” We categorised maternal education into three categories: (1) elementary education, (2) secondary education, including apprentice school, and (3) university education.

We asked about the availability of amenities in the households of respondents [53] using the question: “Is the following in your household? (cold running water, hot running water, working flushing toilet, working bathroom or shower, and electricity)”. The response categories were “Yes” and “No”. We used items covering running water and flushing toilets for the present study.

Since the free movement of stray animals is relatively common in MRCs in contrast to ownership of pets in the households of the majority population, we used the following approach to assess the risk resulting from contact with animals. We asked participants whether they meet animals (dogs, cats, etc.) daily (yes/no) and whether they regularly come into contact with animals (cats, dogs, etc.) that are not regularly checked by a veterinarian (dewormed and vaccinated) (yes/no).

We computed a composite variable of risk factors to assess cumulative risk resulting from regular contact with un-dewormed animals and the absence of running water and flushing toilets in the households.

Health complaints of children in the past month, as reported by mothers, were assessed by asking whether the child suffered from any of the following problems in the last month: (1) headache; (2) stomach ache; (3) cold; (4) flu; (5) cough; (6) constipation; (7) diarrhoea; (8) sadness/tearfulness; (9) irritability/bad mood; (10) fatigue; 11. sleeplessness; 12. loss of appetite. Sum scores were calculated, with a higher score indicating more health complaints.

### Examination of faecal samples

Parasites were assessed in the stool samples of infants. All samples were examined by applying the flotation concentration technique and subsequently examined microscopically for the presence of oocysts, cysts of protozoan parasites and eggs of helminths. Furthermore, qualitative evidence of coproantigen of *Cryptosporidium* spp. was carried out using the ELISA method. Experienced parasitologists performed the morphological characterisation of the propagative parasitic stages following Garcia 2016 [54].

### Statistical analyses

First, we described the demographic and socioeconomic characteristics of the sample and the risk factors of parasitic infections using descriptive statistics. Differences between the two groups (MRCs vs. majority population) were assessed using the Chi-squared.

Next, we used the Chi-squared test to compare the prevalence of intestinal parasitic infections between infants from MRCs and from the majority population. We also compared health complaints of children with and without intestinal parasitic infection in the past month, as reported by mothers. Finally, we assessed the association between risk factors and the occurrence of intestinal parasitic infection in infants from MRCs (infants from the majority were excluded from these analyses) using simple logistic regression on 2,000 bootstrapped samples, with each factor being entered separately into the model to assess the crude associations. A *p*-value lower than 0.05 was considered significant. Statistical analyses were performed using IBM SPSS 23 for Windows and Stata (special edition 14).

## Results

Demographic and socioeconomic descriptions of the sample can be found in Table 1. Statistically significant differences between infants from MRCs and the majority can be seen in the number of siblings, persons per room, and maternal education. Mothers from MRCs significantly more often reported contact with un-dewormed animals and the absence of running water and flushing toilets in the households (Table 1).

**Table 1:** Demographic and socioeconomic description of the sample and risk factors contributing to the occurrence of parasitic infections – comparison between majority and marginalised Roma communities

	Majority *N* = 105		MRCs *N* = 76		Total *N* = 181		*p*-value
	*N*	(%)	*N*	(%)	*N*	(%)	*p*-value
Child’s sex							0.765
boys	46	43.8	35	46.1	81	44.8	
girls	59	56.2	41	53.9	100	55.2	
Education mother							0.000
elementary	2	1.9	60	80.0	62	34.6	
secondary	22	21.2	15	20.0	37	20.7	
university	80	76.9	0	0	80	44.7	
Contact with animals	50	48.1	51	67.1	101	56.1	0.011
Contact with un-dewormed animals	6	6.0	34	46.6	40	23.1	0.000
No running water	0	0	35	46.1	35	19.4	0.000
No flushing toilet	0	0	53	69.7	53	29.4	0.000

### Prevalence of parasitic infections

No protozoan oocysts or cysts, nor helminth eggs, were detected in the group of infants from the majority population. Of 181 samples, 23 were identified as positive, all from the infants living in MRCs (Table 2). The microscopic examination revealed the presence of parasitic infection in infants from MRCs with intestinal protozoan and helminth parasites. Parasitological examination in infants from MRCs (*n* = 76) revealed two significant species of nematodes (*Ascaris lumbricoides, Trichuris trichiura*), one tapeworm (*Hymenolepis nana*), and one protozoan species (*Giardia duodenalis*). Among the protozoan parasite, *G. duodenalis* was found in 9.2% (7/76) of infants from MRCs, while among the most common helminths were *A. lumbricoides* 21.1% (*n* = 19/76), *T. trichiura* was found in 6.6% (*n* = 5/76), and *H. nana* was identified only in one case (*n* = 1/76). Light, moderate and heavy species-specific classes of infection intensity were observed in the samples.

**Table 2:** Intestinal parasitic infections prevalence – comparison between infants from majority and marginalised Roma communities

	Majority *N* = 105		MRCs *N* = 76		Total *N* = 181		*p*-value
	N	(%)	N	(%)	N	(%)	
Positive cases	0	0	23	30.3	23	12.7	0.000
*Giardia duodenalis*	0	0	7	9.2	7	3.9	0.002
*Trichuris trichiura*	0	0	5	6.6	5	2.8	0.008
*Ascaris lumbricoides*	0	0	16	21.1	16	8.8	0.000
*Hymenolepis nana*	0	0	1	1.3	1	0.6	0.239
Polyparasitism status							0.000
single	0	0	18	23.7	18	9.9	
dual	0	0	4	5.3	4	2.2	
triple	0	0	1	1.3	1	0.6	

Single infection was observed in 23.7% (18/76) and mixed in 6.6% (5/76) (Table 2). Details of mixed infections in infants can be seen in Table 3. The prevalence in the infants living in MRCs was statistically higher than in the infants from the majority. Out of 23 positive samples, STHs were found in 17 samples, of which nine were from the same MRC with poor infrastructure where dwellings are surrounded by dirt, soil and mud. In this study, we did not confirm the presence of any coproantigen of *Cryptosporidium* spp. by applying the ELISA method.

**Table 3:** Mixed infections in infants from MRCs

Mixed infection	Giardia duodenalis	Trichuris trichiura	Ascaris lumbricoides	Hymenolepis nana
Case 1	1	1	1	0
Case 2	1	1	1	0
Case 3	0	1	1	0
Case 4	0	1	1	0
Case 5	0	0	1	1

### Health complaints of infants with and without intestinal parasitic infection

Infants with parasitic infections suffer significantly more often from various health complaints compared to infants without such infections. Health complaints of infected infants reported by mothers include headache, stomach ache, cough, diarrhoea, sadness/tearfulness, irritability, and sleeplessness (Table 4). The most common health complaint in infected infants is cough, reported by more than ¾ of mothers of infected infants. Half of the mothers of infected infants also reported stomach aches and irritability. Diarrhoea was reported by 45.5% of mothers.

**Table 4:** Health complaints of children with and without intestinal parasitic infection in the past month as by mothers

	Children without parasitic infection *N* = 157		Children with parasitic infection *N* = 22		Total *N* = 181		*p*-value
	N	(%)	N	(%)	N	(%)	N
headache	5	3.2	4	18.2	9	5.0	0.003
stomach ache	19	12.1	11	50.0	30	16.8	0.000
cold	83	52.9	13	59.1	96	53.6	0.583
flu	18	11.5	5	22.7	23	12.8	0.139
cough	65	41.4	17	77.3	82	45.8	0.002
constipation	8	5.1	1	4.5	9	5.0	0.912
diarrhoea	29	18.5	10	45.5	39	21.8	0.004
sadness, tearfulness	22	14.0	9	40.9	31	17.3	0.002
irritability	43	27.4	11	50.0	54	30.2	0.030
fatigue	19	12.1	4	18.2	23	12.8	0.425
sleeplessness	21	13.4	8	36.4	29	16.2	0.006
loss of appetite	26	16.6	5	22.7	31	17.3	0.474

### Association between risk factors and intestinal occurrence of parasitic infection among children from MRCs

For this analysis, we selected only infants from MRCs. All explored risk factors were found to be associated with intestinal parasitic infection in infants from MRCs, except for the absence of running water in the household. Assessment of the effect of cumulative risk (absence of running water, flushing toilet and contact with un-dewormed animals) showed that two or more risk factors are associated with a higher risk of intestinal parasitic infection in infants from MRCs (Table 5).

**Table 5:** Logistic regression analysis of association between risk factors and occurrence of intestinal parasitic infection in infants from marginalised Roma communities (*N* = 76)

	Crude effect OR (CI)	*p*-value
No running water^a^	1.49 (0.54; 4.12)	0.444
No flushing toilet^a^	4.17 (1.06; 16.40)	0.041
Contact with un-dewormed animals^b^	3.61 (1.11; 11.75)	0.033
Cumulative risk (reference category 0 risk factors)^c^		
1 risk factor	5.60 (0.91; 34.65)	0.064
2 risk factors	8.24 (1.70; 39.80)	0.009
3 risk factors	10.89(1.98; 59.78)	0.006

a = 1 missing value, b = 3 missing values, c = 4 missing values

## Discussion

In the present study, we compared the occurrence of intestinal parasitic infections between infants from the Slovak majority population and from marginalised Roma communities, which are two distinct groups in terms of socioeconomic conditions. We focused on parasitic propagative stages (oocysts, cysts and eggs) in the faeces of children under two years old by applying the flotation method and ELISA test. We explored health complaints of children with and without intestinal parasitic infection in the past month. Finally, we assessed the effect of various risk factors on the occurrence of intestinal parasitic infection in infants from marginalised Roma communities.

Our findings revealed significantly higher infection rates in infants from MRCs with common parasites, including *Ascaris lumbricoides*, *Trichuris trichiura*, and *Giardia duodenalis*. In contrast, no parasitic infections were detected in the infant group from the majority population with middle-to-high socioeconomic status. Similarly, previous studies conducted in Slovakia suggest preschool-aged children from MRCs are the most vulnerable [62–64]. On the contrary, the prevalence of intestinal parasites among children from the Slovak majority population is very low [63–66], which aligns with our results. These results also align with previous research from other countries, highlighting the disproportionate burden of parasitic infections among disadvantaged populations, particularly children living in impoverished conditions [2, 29, 42, 49].

Infected infants from MRCs frequently exhibited symptoms such as cough, stomach ache, irritability, and diarrhoea. This is similar to findings from several studies in which, e.g. diarrhoea is one of the most typical symptoms of giardiasis [55–59], but the asymptomatic course is also possible [60]. In the study by Hamid et al., 42.4% of the examined preschool children had diarrhoea, of which 59% were infected with *H. nana*, and 41% tested negative for the presence of this parasite in stool [44]. Symptoms of pulmonary ascariasis, referred to as Löffler’s syndrome, include chest discomfort (burning sensation intensified by coughing), dry cough, shortness of breath, fever, expectoration (possibly with blood streaks), and wheezing [61]. The efficacy of the immune response may fluctuate based on factors such as the parasite species, the intensity of infection, and the general health condition of infants, which is also influenced by restricted access to healthcare and basic hygiene conditions in MRCs. The combination of these factors can lead to weakened immune systems in children from MRCs and an increased risk of infections overall. Nevertheless, parasitic infections among children can lead to a variety of health complaints, underscoring the importance of effective prevention and treatment strategies to mitigate the adverse effects on pediatric health and well-being.

We also found a significant association between multiple risk factors, such as lack of access to sanitation and contact with un-dewormed animals, and increased risk of parasitic infections in infants from MRCs. This underscores the critical importance of addressing socioeconomic disparities and improving access to basic sanitation and healthcare services in MRCs to reduce the prevalence of parasitic infections and mitigate their adverse health effects. The spread of STHs occurs worldwide, wherever environmental conditions are suitable for completing the life cycle of these parasites in the soil. In MRCs, paved or grassed areas and sidewalks are often lacking, and the surroundings of dwellings consist only of raw soil. This increased risk of contact with soil, where cysts of *Giardia* and eggs of STHs can survive for several months, in the case of *Ascaris lumbricoides*, even years, particularly exposes children to the risk of infection. Maintaining hygienic standards in conditions of socioeconomic deprivation in MRCs without running water and sewage is problematic. Moreover, the environment of MRCs is associated with a significant number of dogs infected with parasites and greater soil contamination with propagative stages of parasites [62–65]. Additionally, a lack of awareness in MRCs heightens the risk of infection spreading. All the factors mentioned above then contribute to an increased risk of reinfection of children despite anthelmintic treatment once they return to the same environment.

The intensity of infection spread depends on socioeconomic and regional geoclimatic factors, primarily on population density, education, hygiene conditions, and dietary habits. The level of personal and communal hygiene is reflected in the level of environmental contamination with helminth pathogens and the occurrence of helminth infections in domestic animals and inhabitants, especially in children. Several studies have shown that soil is a significant source of infections, which notably impacts public health [67, 68]. Children stand as the most susceptible group, influenced by various risk factors like generational poverty, insufficient education, and restricted or absent access to sanitation and clean water. The factors associated with the onset of the parasitic disease include the age and clinical, nutritional, and immune status of the infected individual. In poor socioeconomic and hygienic conditions, people of all age groups experience a cycle of repeated infections, particularly children [69, 70].

### Strengths and limitations

The present study provides evidence of persisting early childhood inequities. It demonstrates health disparities between children from MRCs and the majority, considering parasitic infections and risk factors contributing to unfavourable health outcomes. Among the limitations that need to be mentioned is the cross-sectional design, which does not allow conclusions about causality. Sample size and the representativeness of the population studied may not fully capture the diversity within the MRCs, potentially affecting the generalizability of the findings to communities characterised by spatial and social distance from the majority population. Furthermore, self-reported data are prone to social desirability, which may cause an underestimation of the prevalence of health risks as well as health complaints of infants.

### Implication

Our study highlights the disproportionate burden of parasitic infections among infants from MRCs, driven by socioeconomic disparities and environmental risk factors. Addressing the lack of basic sanitation, such as running water and flushing toilets, in MRCs is crucial for reducing the risk of parasitic infections among infants. Investments in sanitation infrastructure can significantly improve health outcomes and reduce the burden of preventable diseases. There is also a need for targeted health education campaigns to raise awareness about transmitting, preventing, and treating parasitic infections within MRCs. Empowering caregivers with knowledge about hygienic practices, including handwashing and proper waste disposal, can help mitigate the risk of infection spread.

The health complaints reported by mothers of infected infants underscore the impact of parasitic infections on child health and well-being, highlighting the need for targeted interventions to address this public health challenge. Ensuring equitable access to healthcare services, including regular screening and treatment for parasitic infections, is essential for addressing the health disparities observed in MRCs. Outreach programs and mobile health clinics can help overcome barriers to access and reach vulnerable populations.

Parasitic infections represent a burden not only for individual health but also on the public health level. Efforts to reduce environmental contamination with parasitic pathogens, such as deworming programs for domestic animals and soil decontamination initiatives, can contribute to breaking the cycle of infection transmission within MRCs. Addressing the complex health challenges MRCs face requires coordinated efforts involving collaboration between health authorities, social services, education agencies, and community-based organisations.

## Conclusion

Our study underscores the disproportionate burden of parasitic infections among infants from marginalised Roma communities (MRCs) in Slovakia, driven by socioeconomic disparities and environmental risk factors. The living conditions in MRCs, characterised by a lack of running water, sewage systems, and exposure to un-dewormed animals, contribute to the higher prevalence of parasitic infections in children from these communities. These infections pose significant health risks and threaten the healthy development of affected children. Improving sanitation infrastructure, promoting health education, and expanding access to healthcare services are essential in reducing the prevalence of parasitic infections and improving health outcomes among infants in MRCs. Additionally, environmental interventions, such as castration programs to reduce the number of contaminated animals, can be crucial in limiting the spread of parasites with zoonotic potential. Addressing the underlying determinants of health disparities and implementing evidence-based interventions are essential steps towards achieving health equity and ensuring that all children have the opportunity to thrive, regardless of their socioeconomic background or place of residence.

### Electronic supplementary material

Below is the link to the electronic supplementary material.

Supplementary Material 1

## Data Availability

Data are available from the authors upon reasonable request.

## Abbreviations

MRCs: marginalised Roma communities
STHs: Soil-transmitted helminths
A. lumbricoides: Ascaris lumbricoides
T. trichiura: Trichuris trichiura
G. duodenalis: Giardia duodenalis
H. nana: Hymenolepis nana

## References

[1] Lustigman S, Prichard RK, Gazzinelli A, Grant WN, Boatin BA (2012). A Research Agenda for Helminth diseases of humans: intervention for control and elimination. PLOS Negl Trop Dis.

[2] WORLD HEALTH ORGANIZATION (WHO). Soil-transmitted helminth infections. [online]. 2020, [cit. 2020-08-15]. Available on Internet: <https://www.who.int/news-room/fact-sheets/detail/soil-transmitted-helminth-infections

[3] EU FRA - European Union Agency for Fundamental Rights (2022). Roma in 10 European countries: main results. Roma survey 2021.

[4] Vacková J, Maňhalová J, Rolantová, Urban D. Health literacy in the Roma population. KONTAKT-Journal Nurs Social Sci Relat Health Illn 2020; 22(4).

[5] Hajare ST, Gobena RK, Chauhan NM, Eriso F (2021). Prevalence of intestinal parasite infections and their associated factors among food handlers working in selected catering establishments from Bule Hora, Ethiopia. Biomed Res Int.

[6] Wakid MH (2009). Intestinal parasitic infection among Food handlers in Holy City Makkah during Hajj season 1428 Hegira 2007. Med Sci.

[7] WORLD HEALTH ORGANIZATION (WHO). Soil-transmitted helminth infections. [online]. 2024, [cit. 2020-04-22].Available on Internet: <https://www.who.int/news-room/fact-sheets/detail/soil-transmitted-helminth-infections

[8] WORLD HEALTH ORGANIZATION (WHO). Zoonoses. [online]. 2020, [cit. 2020-12-08]. Available on Internet: <https://www.who.int/zoonoses/en/

[9] Xiao L, Feng Y (2017). Molecular epidemiologic tools for waterborne pathogens Cryptosporidium spp. and Giardia Duodenalis. Food Waterborne Parasitol.

[10] Certad G, Viscogliosi E, Chabé M, Cacció SM (2017). Pathogenic mechanisms of Cryptosporidium and Giardia. Trends Parasitol.

[11] Allain T, Buret AG (2020). Pathogenesis and post-infectious complications in giardiasis. Adv Parasitol.

[12] Niehaus MD, Moore SR, Patrick PD, Derr LL, Lornitz DB, Lima AA (2002). Early childhood diarrhea is associated with diminished cognitive function 4 to 7 years later in children in a northeast Brazilian shantytown. ASTMH.

[13] Plutzer J, Ongerth J, Karanis P (2010). Giardia taxonomy, phylogeny and epidemiology: facts and open questions. J Hyg Environ.

[14] Dayao A, Sheoran A, Carvalho A, Xu H, Beamer G, Widmer G (2020). An immunocompetent rat model of infection with Cryptosporidium hominis and Cryptosporidium parvum. Int J Parasitol.

[15] Putignani L, Menichella D (2010). Global distribution, public health and clinical impact of the protozoan pathogen cryptosporidium. Interdiscip Perspect Infect Dis.

[16] Khalil IA, Troeger C, Rao PC, Blacker BF, Brown A, Brewer TG (2018). Morbidity, mortality, and long-term consequences associated with diarrhoea from Cryptosporidium infection in children younger than 5 years: a meta-analyses study. Lancet Glob Health.

[17] Pumipuntu N, Piratae S, Cryptosporidiosis. A zoonotic disease concern. Vet World. 2018;5681–6. 10.14202/vetworld.2018.681-686 10.14202/vetworld.2018.681-686 PMC5993756 29915508

[18] Ahmed M (2023). Intestinal parasitic infections in 2023. Gastroenterol Res.

[19] Bartsch SM, Hotez PJ, Asti L, Zapf KM, Bottazzi ME, Diemert DJ, et al. The global economic and health burden of human hookworm infection. PLoS Negl Trop Dis. 2016;e0004922. 10.9. 10.1371/journal.pntd.0004922 PMC5015833 27607360

[20] Weatherhead JE, Hotez PJ, Meija R (2017). The global state of helminth control and elimination in children. Clin Pediatr.

[21] Zammarchi L (2017). Spectrum and burden of neglected tropical diseases observed in an infectious and tropical diseases unit in Florence, Italy (2000–2015). Intern Emerg Med.

[22] Bethony J, Brooker S, Albonico M, Geiger SM, Loukas A, Diemert D (2006). Soil-transmitted helminth infections: ascariasis, trichuriasis, and hookworm. Lancet.

[23] Khuroo MS, Rather AA, Khuroo NS, Khuroo MS (2016). Hepatobiliary and pancreatic ascariasis. World J Gastroenterol.

[24] Gelanew T, Lalle M, Hailu A, Pozio E, Cacció SM (2007). Molecular characterisation of human isolates of Giardia Duodenalis from Ethiopia. Acta trop.

[25] Khan W, Rahman H, Rafiq N, Kabir M, Ahmed MS, Escalante PLR. Risk factors associated with intestinal pathogenic parasites in schoolchildren. Saudi J Biol Sci. 2022;42782–6. 10.1016/j.sjbs.2021.12.055 10.1016/j.sjbs.2021.12.055 PMC9072892 35531160

[26] Benjamin-Chung J, Mertens A, Colford JM (2023). Early-childhood linear growth faltering in low- and middle-income countries. Nature.

[27] Olopade BO, Idowu CO, Oyelese AO, Aboderin AO (2018). Intestinal parasites, nutritional status and cognitive function among primary school pupils in ile-ife, Osun state, Nigeria. Afr J Infect Dis.

[28] Bogitsh BJ, Carter CE, Oeltmann TN. Human parasitology. 4th Edition. Oxfort, UK: Elsevier, 2013, 430, ISBN 978-0-12-415915-0.

[29] Deplazes P, Eckert J, Mathis A, Samson-Himmelstjerna G, Zahner H. Parasitology in Veterinary Medicine. The Netherlands, 2016, 650, ISBN 97890-8686-274-0.

[30] Dickson R, Awasthi S, Williamson P, Demellweek C, Garner P (2000). Effects of treatment for intestinal helminth infection on growth and cognitive performance in children: systematic review of randomised trials. BMJ.

[31] Galgamuwa LS, Iddawela D, Dharmaratne SD (2018). Prevalence and intensity of Ascaris lumbricoides infections in relation to undernutrition among children in a tea plantation community, Sri Lanka: a cross-sectional study. BMC Pediatr.

[32] De Lima Corvino DF, Horrall S. Ascariasis. Stat Pearls (Internet). 2022. https://www.ncbi.nlm.nih.gov/books/NBK430796/28613547

[33] Khalil IA, Troeger C, Rao PC, Blacker BF, Brown A, Brewer TG (2018). Morbidity, mortality, and long-term consequences associated with diarrhoea from Cryptosporidium infection in children younger than 5 years: a meta-analyses study. Lancet Glob Health.

[34] Mohd-shaharuddin N, Lian Lim YA, Ngui R, Nathan S (2021). Expression of Ascaris lumbricoides putative virulence-associated genes when infecting a human host. Parasit Vectors.

[35] Phuphisut O, Poodeepiyasawat A, Yoonuan T, Watthanakulpanich D, Chotsiri P, Reamtong O, Mousley A, Gobert GN, Adisakwattana P (2022). Transcriptome profiling of male and female Ascaris lumbricoides reproductive tissues. Parasit Vectors.

[36] Kache S (2020). COVID-19 PICU guidelines: for high-and limited-resource settings. Pediatr Res.

[37] Lim-leroy A, CHua TH (2020). Prevalence and risk factors of geohelminthiasis among the rural village children in Kota Marudu, Sabah, Malaysia. PLoS ONE.

[38] Pullan RL, Brooker SJ (2012). The global limits and population at risk of soil-transmitted helminth infections in 2010. Parasit Vectors.

[39] Else KJ, Keiser J, Holland CV, Grencis RK, Sattelle DB, Fujiwara RT, Bueno LL, Asaolu SO, Sowemimo OA, Cooper PJ. Whipworm and roundworm infections. Nat Rev Dis Primers. 2020; 28;6(1):44. 10.1038/s41572-020-0171-3. PMID: 32467581. 10.1038/s41572-020-0171-3 32467581

[40] Brustoloni YM, Chang MR, Lyrio de Oliveira AM, Silva de Alexandre A (2009). Trichiura eggs found in oral mucosal lesions in a child in Brazil. Parasitol Int.

[41] Khuroo MS, Khuroo MS, Khuroo NS (2010). Trichuris dysentery syndrome: a common cause of chronic iron deficiency anemia in adults in an endemic area (with videos). Gastrointest Endoscopy.

[42] Bundy DA, Cooper ES, Thompson DE, Didier JM, Simmons I (1987). Epidemiology and population dynamics of Ascaris lumbricoides and Trichuris trichiura infection in the same community. Trans R Soc Trop Med Hyg.

[43] Kaminsky RG, Castillo RV, Flores CA (2015). Growth retardation and severe anemia in children with Trichuris dysenteric syndrome. Asian Pac J Trop Biomed.

[44] Hamid MMA, Eljack IB, Osman MKM, Elaagip HA, Muneer MS (2015). The prevalence of Hymenolepis nana among preschool children of displacement communities in Khartoum state, Sudan: a cross-sectional study. Travel Med Infec Dis.

[45] Dahmana H, Granjon L, Diagne C, Davoust B, Fenollar F, Mediannikov O (2020). Rodents as hosts of pathogens and related zoonotic disease risk. Pathogens.

[46] Jarošová J, Antolová D, Šnábel V, Miklisová D, Cavallero S (2020). The dwarf tapeworm Hymenolepis Nana in Pet rodents in Slovakia—epidemiological survey and genetic analysis. Parasitol Res.

[47] Dold Ch, Holland CV (2011). Ascaris and ascariasis. Microbes Infect.

[48] Drake LJ, Jukes MCH, Sternberg RJ, Bundy DAP. Geohelminth infections (ascariasis, trichuriasis, and hookworm): cognitive and developmental impacts. Semin Pediatr Infect Dis. 2000;245–51. 10.1053/spid.2000.9638

[49] GBD. Mortality and Causes of Death Collaborators (2016). Global, regional, and national life expectancy, all-cause mortality, and cause-specific mortality for 249 causes of death, 1980–2015: a systematic analysis for the global burden of Disease Study 2015. Lancet.

[50] Tchuem Tchuenté LA (2011). Control of soil-transmitted helminths in sub-saharan Africa: diagnosis, drug efficacy concerns and challenges. Acta Trop.

[51] Ministry of the Interior of Slovak Republic. Atlas of Roma communities. (2019). http://www.minv.sk/?atlas-romskych-komunit-2019. March 20, 2022.

[52] Kolarcik P, Madarasova Geckova A, Orosova O, van Dijk JP, Reijneveld SA (2010). Predictors of health-endangering behaviour among Roma and non-roma adolescents in Slovakia by gender. JECH.

[53] Madarasova Geckova A, Babinska I, Bobakova D, Dankulincova Veselska Z, Bosakova L, Kolarcik P (2014). Socioeconomic characteristics of the Population Living in Roma Settlements and Their Association with Health and Health-related Behaviour. CEJPH.

[54] Garcia. 2016.

[55] Cotton JA, Beatty JK, Buret AG (2011). Host parasite interactions and pathophysiology in Giardia infections. Int J Parasitol.

[56] Gelanew T, Lalle M, Hailu A, Pozio E, Cacció SM (2007). Molecular characterisation of human isolates of Giardia Duodenalis from Ethiopia. Acta trop.

[57] Perruccci S, Berrilli F, Procopio C, Di Filippo MM, Pierini A, Marchetti A (2020). Giardia duodenalis infection in dogs affected by primary chronic enteropathy. Open Vet J.

[58] Scorza AV, Ballweber LR, Tangtrongsup S, Panuska C, Lappin MR (2012). Comparisons of mammalian Giardia duodenalis assemblages based on the β-giardin, glutamate dehydrogenase and triose phosphate isomerase genes. Vet Parasitol.

[59] Homan WL, Mank, Theo G (2001). Human giardiasis: genotype linked differences in clinical symptomatology. Int J Parasitol.

[60] Muadica AS, Koster PC, Dashti A, Bailo B, Hernandéz de Mingo M, Reh L (2020). Molecular diversity of Giardia Duodenalis, Cryptosporidium spp. and Blastocystis sp. in asymptomatic school children in Leganés, Madrid (Spain). Microorganism.

[61] Łanocha A, Lanocha –Arendarczyk N, Wilczýnska D, Zdziarska B (2022). Kosik –Bogacka D. Protozoan intestinal parasitic infection in patients with hematological malignancies. J Clin Med.

[62] Rudohradská P (2012). Prevalence of intestinal parasites in children from minority group with low hygienic standards in Slovakia. Helminthologia.

[63] Štrkolcová G, Goldová M, Bocková E, Mojžišová J (2017). The roundworm Strongyloides Stercoralis in children, dogs, and soil inside and outside a segregated settlement in Eastern Slovakia: frequent but hardly detectable parasite. Parasitol Res.

[64] Štrkolcova G, Mravcová K, Barbušinová E, Mucha R, Várady M, Goldová M. Prevalence of intestinal parasites in Children living in various living conditions in Slovakia. J Paediatr Child Health. 2019;174–85. 10.26502/jppch.74050028

[65] Pipíkova J, Papajová I, Šoltýs J, Schusterová I, Kočišová D, Tothayová A (2017). Segregated settlements present an increased risk for the parasite infections spread in Northeastern Slovakia. Helminthologia.

[66] Šmigová J, Šnábel V, Cavallero S, Šmiga Ľ, Šoltys J, Papaj J, Papajová I (2022). Neglected diseases-parasitic infections among Slovakian children from different populations and genotypes of Giardia Duodenalis. Microorganisms.

[67] Giacometti A, Cirioni O, Fortuna M, Osimani P, Antonicelli L, Del Prete MS (2000). Environmental and serological evidence for the presence of toxocariasis in the urban area of Ancona, Italy. Eur J Epidemiol.

[68] Martínez-Moreno FJ, Hernandéz S, López-Cobes E, Becerra C, Acosta L, Martinéz- Moreno A (2007). Estimation of canine intestinal parasites in Cordoba (Spain) and their risk to public health. Vet Parasitol.

[69] Garzon DL (2005). Contributing factors to preschool unintentional injury. J Pediatr Nurs.

[70] Steketee RW (2003). Pregnancy, nutrition and parasitic diseases. Nutr J.

