# Supplementary material for: Intestinal parasitic infections in children from marginalised Roma communities: prevalence and risk factors
Source: BMC Infect Dis. 2024 Jun 18;24:596. doi: 10.1186/s12879-024-09500-z (PMC11184866; doi:10.1186/s12879-024-09500-z)
Supplement: Supplementary file 1 — Supplementary Material 1 [file 12879_2024_9500_MOESM1_ESM.docx]

**Appendix 1**

| 1. What education have you completed? | |
| --- | --- |
| 🞎 | Elementary (completed or uncompleted) |
| 🞎 | Vocational secondary school (such as mason, electrician) |
| 🞎 | Specialised secondary school or grammar school |
| 🞎 | University (title Bc., MSc., MD., JD., etc.) |
| 🞎 | I don’t know |

| 1. Are your closest neighbours mostly Roma? | 🞎 | Yes | 🞎 | No |
| --- | --- | --- | --- | --- |

| 1. Is the following in your household? | |
| --- | --- |
| 🞎 | Cold running water |
| 🞎 | Hot running water |
| 🞎 | Working flushing toilet |
| 🞎 | Working bathroom or shower |
| 🞎 | Electricity |

| 1. Has your child suffered from any of the following problems in the past month? | |
| --- | --- |
| 🞎 | Headache |
| 🞎 | Stomach ache |
| 🞎 | Cold |
| 🞎 | Flu |
| 🞎 | Sadness, tearfulness |
| 🞎 | Irritability and/or bad mood |
| 🞎 | Cough |
| 🞎 | Fatigue |
| 🞎 | Sleeplessness |
| 🞎 | Loss of appetite |
| 🞎 | Constipation |
| 🞎 | Diarrhoea |
| 🞎 | Allergy |
| 🞎 | Other, specify............................................................ |

| 1. Do you meet animals (dogs, cats, etc.) daily? | 🞎 | Yes | 🞎 | No |
| --- | --- | --- | --- | --- |

| 1. Do you regularly come into contact with animals (cats, dogs, etc.) that are not regularly checked by a veterinarian (dewormed and vaccinated)? | |
| --- | --- |
| 🞎 | Yes |
| 🞎 | No |
| 🞎 | I don’t know |
